# Pregnancy outcome treated with stage-by-stage acupuncture and moxibustion therapy based on the chong channel being sea of blood theory in repeated IVF-ET failure patients: A randomized controlled trial

**DOI:** 10.1097/MD.0000000000023234

**Published:** 2020-11-20

**Authors:** Liwei Xing, Jinlong Xu, Qiong Zhang, Li Li, Yunxiu Li, Haina Zhuang, Zhuojun Yuan, Rong Zhao, Yanping Ma

**Affiliations:** a Yunnan University of Traditional Chinese Medicine; b Yunnan Second People's Hospital; c Yunnan First People's Hospital; d Yunnan Provincial Hospital of Traditional Chinese Medicine, Kunming, Yunnan province, China.

**Keywords:** acupuncture, endometrial receptivity, IVF-ET, pregnancy outcome, RIF, stage-by-stage acupuncture and moxibustion therapy

## Abstract

**Introduction:**

Acupuncture and moxibustion has become a commonly used adjuvant treatment method to improve the success rate of in vitro fertilization-embryo transfer (IVF-ET). However, There is still insufficient evidence that acupuncture treatment can improve the local microenvironment of endometrium, the endometrial receptivity, and the pregnancy outcome of patients, which is worthy of further study.

**Method/Design:**

To investigate the effect of Stage by Stage Acupuncture and Moxibustion Therapy on endometrial receptivity and Pregnancy Outcome based on the theory of “Chong channel being sea of blood,” we will conduct a multicenter randomized controlled trial. Inclusion criteria are as follows: infertile women under 45 years of age who received IVF-ET or Intracytoplasmic sperm injection cycles. The study will only be applied to women who have failed repeated implantation, that is, women who have failed 3 or more embryo transplants in the past (existing frozen embryos do not require the retrieval of eggs). Those who are not prepared to receive IVF-ET or are at risk of pregnancy, have a serious medical condition, or are egg donors will be excluded. Subjects will be randomly assigned to either the acupuncture group (IVF-ET plus stage-by-stage acupuncture and moxibustion therapy based on the “Chong channel being sea of blood” theory) or the control group (IVF-ET only). The trial required a total sample size of 246 women to compare endometrial receptivity between the 2 groups. The acupuncture group will receive acupuncture and moxibustion treatment 3 times a week starting from the third day of menstruation in the ovary stimulation cycle. One menstrual cycle was one course of treatment, and a total of 3 menstrual cycles were treated. The main outcome indicator was clinical pregnancy rate. Secondary outcome indicators were the three-dimensional volume blood flow parameters (vascularization index, flow index, and vascularization flow index) of the endometrium, endometrial thickness, endometrial volume, uterine artery PI, RI, and S/D during the “implantation window period” (20–24 days after menstruation in the ovary stimulation cycle).

**Discussion:**

This study will provide important evidence for the use of Stage by Stage Acupuncture and Moxibustion Therapy Based on the “Chong Channel Being Sea of Blood” Theory in IVF.

**Trial registration:**

http://www.chictr.org.cn/edit.aspx?pid=28811&htm=4 ID: ChiCTR1800017191 (07/17/2018).

## 1 Introduction

Infertility, together with malignant tumors and cardiovascular diseases, has been listed as 1 of the 3 major diseases affecting the quality of human life.^[1]^ With the opening up of China's 2-child policy and the continuous increase of fertility requirements, the number of elderly mothers is increasing, infertility has become a serious social problem.^[2]^ In vitro fertilization—embryo transfer has become the most effective treatment of infertility. Although in vitro fertilization-embryo transfer (IVF-ET) has been in operation for nearly 30 years since the birth of the world's first IVF baby in 1978, the clinical pregnancy rate remains at only 30%.^[3]^ The latest research shows that two-thirds of the reasons for implantation failure of IVF-ET embryos are due to insufficient endometrial receptivity, endometrial receptivity is closely related to embryo implantation.^[4]^ For IVF-ET patients who failed for 3 times or more, improving endometrial blood perfusion to create a microenvironment suitable for embryo implantation is the most effective way to increase clinical pregnancy.^[5]^ In recent years, acupuncture has shown a good momentum in the application of IVF-ET. The latest meta-analysis shows that acupuncture or acupuncture combined with other therapies can improve the endometrial receptivity of IVF-ET patients.^[6]^ However, the existing researches on acupuncture and moxibustion are generally of heterogeneity, especially in the aspects of acupuncture and moxibustion treatment methods and acupoint selection, currently there is no recognized treatment reference standard, which makes the clinical effect very different,^[7]^ and there are too many studies on acupuncture and moxibustion combined with other therapies to clearly prove the efficacy of acupuncture and moxibustion. In the 19 years of clinical practice, we have formed the stage-by-stage acupuncture and moxibustion therapy for the treatment of female infertility. Previous studies have found that stage-by-stage acupuncture and moxibustion therapy based on the “Chong channel being sea of blood” Theory can increase the thickness of the endometrium in the middle luteal stage of IVF-ET patients with repeated failure of transplantation, and improve the clinical pregnancy rate.^[8]^ Preliminary experiments have shown that the acupuncture therapy can improve the blood flow parameters under the endometrium. To this end, we plan to conduct a more rigorous large-scale multicenter randomized controlled trial to further evaluate the effectiveness of stage-by-stage acupuncture and moxibustion therapy based on the “Chong channel being sea of blood” theory to improve endometrial receptivity and pregnancy outcome.

## 2 Method/design

**Figure 1:**
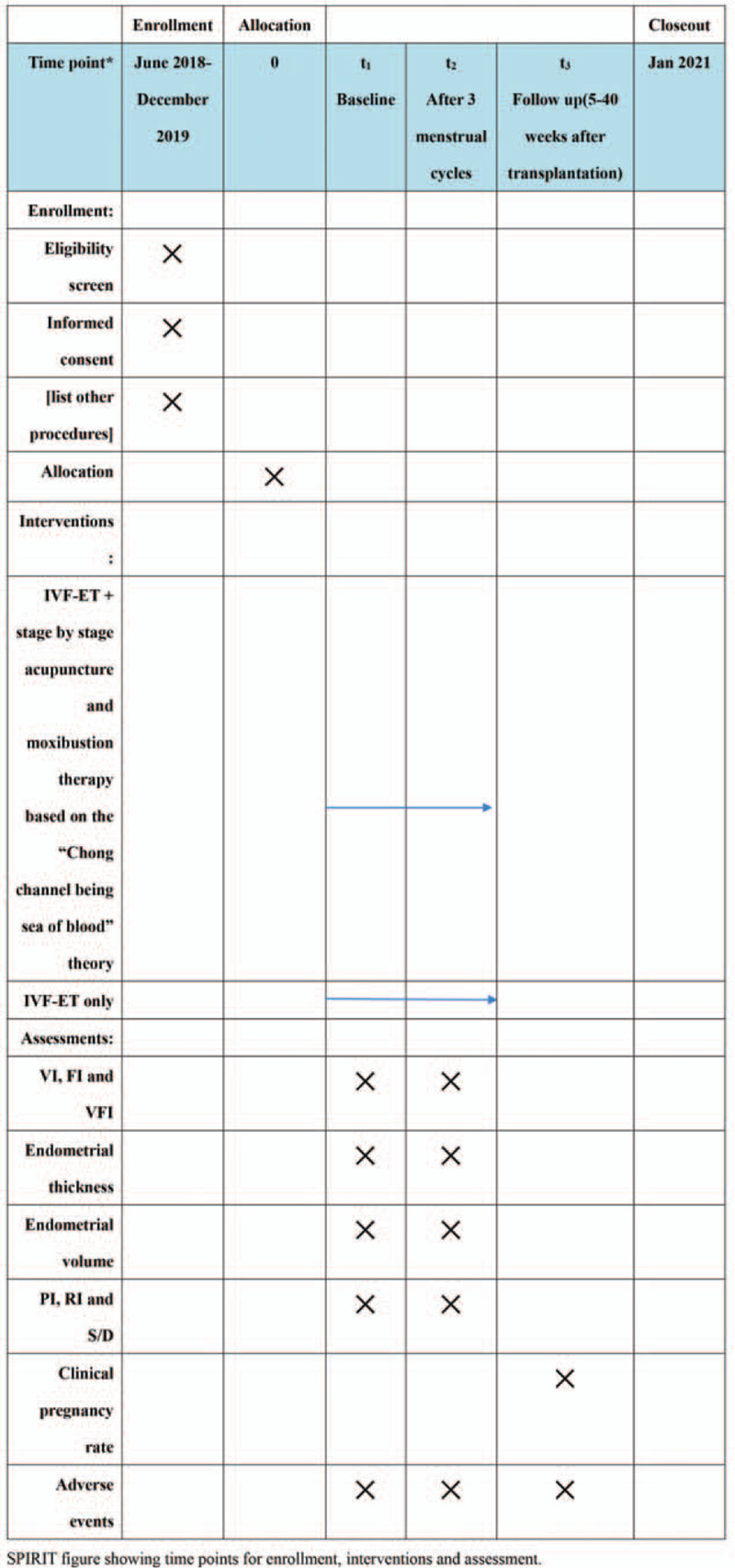
The SPIRIT figure of enrollment, interventions, and assessments. SPIRIT = indicates standard protocol items recommendations for interventional trials.

This study was designed as a multicenter, randomized, parallel controlled, double-blind trial of evaluators and statisticians. Participants will include the following 3 hospitals: Yunnan first people's hospital, Yunnan second people's hospital, and Yunnan provincial hospital of traditional Chinese medicine. Every research center must strictly follow the inclusion and exclusion criteria. The study will be conducted in the following order: patients will be screened and qualified for enrollment, they will be randomly assigned to receive acupuncture 3 times a week, 1 menstrual cycle for 1 course of treatment, a total of 3 menstrual cycles, and a 40-week follow-up period. The research plan has already been clinical trials of traditional Chinese medicine hospital ethics committee approval in Yunnan province, China (grant no. (2017) (032)—01), and registered on the Chinese clinical trial registry (http://www.chictr.org.cn/, ID: ChiCTR1800017191). This study will adhere to the Standard Protocol items: Recommendations for Interventional Trials (SPIRIT) 2013 statement(see Fig. 1 for the SPIRIT figure of enrollment, interventions, and assessments). Every participant will sign an informed consent form. Patients will be enrolled in the study and will be recruited only once and will not receive any financial compensation for their participation in the study.

### 2.1 Eligibility criteria

Women admitted to the reproductive medicine center of the research hospital will be screened to determine their suitability for in vitro fertilization. All IVF indications for women, such as tubal infertility, severe male factor infertility, endometriosis, unexplained infertility, immune infertility, and anovulation infertility, were recommended for this study. The included patients must be infertile women under the age of 45 who have undergone in vitro fertilization-embryo transfer or Intracytoplasmic sperm injection cycles, who have experienced 3 or more IVF-ET failures, and who have frozen embryos and no longer need to take eggs. Exclusion criteria were severe physical diseases or contraindications to acupuncture and moxibustion, poor pregnancy and birth history, thrombotic diseases, etc.

Patients have the right to refuse to participate in the study, and they can withdraw at any time. Their consent or withdrawal would not affect their intended IVF treatment.

### 2.2 Sample size

Based on the calculation of sample size, 2 groups are proposed in this scheme. Test level α=0.05, test efficiency 1−β= 0.90. The retention rate of acupuncture and moxibustion group was based on the effective rate of acupuncture in stages on endometrial receptivity in our previous research results (27%),^[8]^ and the effective rate of subendometrial blood flow parameters in the study of acupuncture in enhancing endometrial receptivity (26.3%) in Hu et al,^[9]^ which was estimated to be 27%. In the meta-analysis based on Smith et al, the effective rate of the blank group was 10%.^[10]^ Therefore, 10% of the control group was selected as the minimum clinical test difference that might change clinical practice. In the staged acupuncture group, 27% was the smallest difference in clinical detection that could change clinical practice, and the shedding rate was 10%. According to the formula $n=\frac{1}{\left(1-f\right)}*\left[\frac{2*{\left(z_{\alpha }+z_{\beta }\right)}^{2}*p*(1-p)}{\left(p_{0}-p_{1}\right)}\right],\;\;\;\;\;p=\frac{\left(p_{0}-p_{1}\right)}{2}$,^[11]^ where n= the sample size of each group. F = shedding rate, value 0.1. P0 = effective rate of the control group, with a value of 0.1. P1 = effective rate in the stage acupuncture group, with a value of 0.27. Z_α_ = 1.65 when α=0.05 (unilateral). When 1−β = 0.90, Z _β_ = 1.28. After calculation, n = 122.12≈123. Finally, the sample size of each group was taken as 123 cases.

### 2.3 Recruitment and registration methods

At present, a large number of infertility patients are seeking treatment in the Yunnan First People's Hospital, Yunnan Second People's Hospital, and Yunnan Provincial Hospital of Traditional Chinese Medicine. Patients will participate in the trial mainly through the recruitment poster of the reproductive center of the relevant hospital.

Patients of interest will be screened by specific researchers who are familiar with the inclusion and exclusion criteria for the study. Participants will have a clear and comprehensive understanding of the trials they will participate in, such as the benefits they may gain, the potential risks, the setup of the trial group, and the interventions they will undertake. We will pay particular attention to baseline characteristics such as age, cause and duration of infertility, ethnic distribution, history of smoking or drinking, history of exposure to radiation or chemotherapy, repeated IVF implantation failure, endometrial disease (inflammation, ectopic), and previous pelvic surgery. Patients who are willing to participate and meet the eligibility criteria will be referred to the acupuncture and moxibustion therapists participating in the study.

### 2.4 Principles of randomness and blindness

#### 2.4.1 Randomly assigned

This is a multicenter, randomized, controlled trial in 3 Chinese hospitals. The central randomization was carried out by Yunnan Provincial Hospital of traditional Chinese medicine, and the pseudo-random numbers were generated by “computer pseudo-random number generation method.” Then the patients represented by the random numbers in the even-numbered position in the sorting list were included in the acupuncture and moxibustion group, and the rest were included in the control group. Two hundred forty-six patients who met the inclusion criteria were randomly divided into 123 patients in the control group (IVF-ET only) and 123 patients in the acupuncture group (IVF-ET plus stage-by-stage acupuncture and moxibustion therapy based on the “Chong channel being sea of blood” theory). Randomized assignments to the acupuncture or control groups will be generated by independent researchers using computer software. Computer-generated treatment codes will be placed in sealed, opaque envelopes, and distributed by dedicated study nurses, who will be trained before the trial and will not participate in treatment or care. The basic principles of randomization are as follows: the researchers cannot predict the allocation of patients, and there will be no change in the allocation after randomization.

#### 2.4.2 The principle of blinded

The evaluator will evaluate the results after the end of the trial, in which the evaluator cannot be the acupuncturist performing the operation, and until the end of the trial, the evaluator does not know the group distribution. Data statisticians are also ignorant of the distribution of groups before making statistical data. The trial will fully follow the blind method, but patients and acupuncturists cannot follow the blind method because there is no sham acupuncture group in the control group.

#### 2.4.3 In vitro fertilization procedure

All selected patients will be treated with the GnRH-a/r-FSH /HMG/HCG long agonist protocol.

On the 21st day of menstruation, a 1-time intramuscular injection of long-acting Tripraline (French Signes) 1.1 to 1.50 mg will be given to achieve downregulation. FSH, LH, and estradiol levels will be measured by fasting blood sampling after 14 days of downregulation. After reaching the downregulation standard (B ultrasound monitoring follicle diameter < 5 mm; endometrial thickness <5 mm; LH < 5 mIU/mL, estradiol < 50 pg/mL), the treatment with gonadotropin (Gn) (Gonafen, 75 IU/ dose, made by Merck serono) for 125 to 300IU superovulation will begin. Gn dosage was determined according to the number of basal sinus follicles, the level of basal sex hormone, and the hormone level of 14 days. Gn was discontinued and HCG (produced by Zhuhai Lizhu Biological Pharmaceutical Co, LTD, Room 405, Administration Building, No.38 chuangye North Road, Jinwan District, Zhuhai City, Guangdong Province, China) was intrusculated 5000 to 10,000 units when there was at least 1 follicle with a > diameter of 18 mm, or 2 or 3 follicle with a > diameter of 17 mm. After the injection of HCG for 34 to 36 hours, the eggs were obtained by vaginal puncture under the guidance of b-ultrasound.

#### 2.4.4 Acupuncture intervention

**Figure 2:**
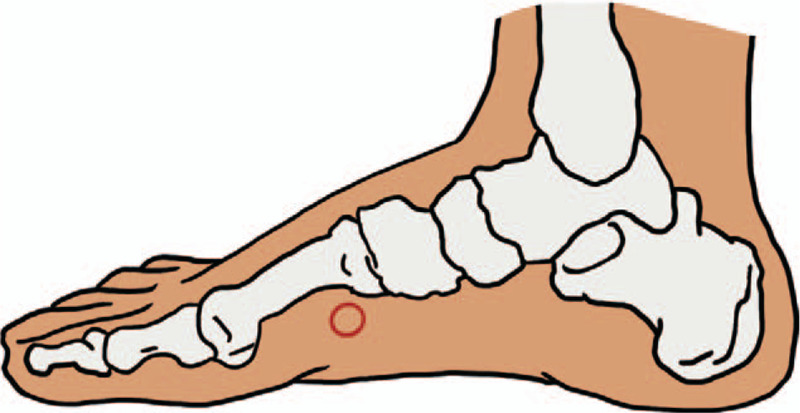
SP-4 (Gongsun) location.

**Figure 3:**
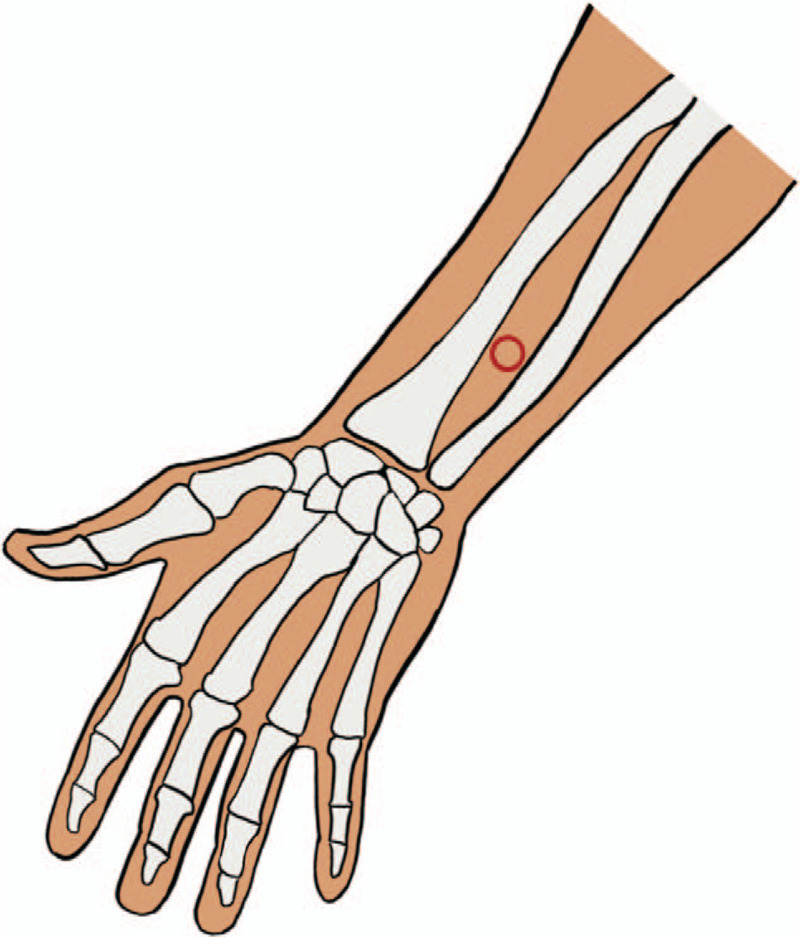
PC-6 (Neiguan) location.

Acupuncture and moxibustion group: Gn RH-a/r-FSH /HMG/HCG agonist long scheme plus Stage by Stage Acupuncture and Moxibustion Therapy Based on the “Chong Channel Being Sea of Blood” Theory. Treatment principle: Adjust qi and blood of Chong channel, because the abundance of Ren channel and Chong channel is a necessary condition for “pregnancy,” which is closely related to the uterus.^[11]^ We mainly use the Chong channel acupoints. Acupuncture prescription: Main acupoints: SP-4(Gongsun) (see Fig. 2), PC-6(Neiguan) (see Fig. 3). Acupoints matching: Add relevant acupoints according to menstrual cycle (follicular phase, ovulation phase, luteal phase) and type differentiation.

### 2.5 Intervention plan

Course of treatment: Acupuncture treatment 3 times a week, 1 menstrual cycle for a course of treatment, a total of 3 menstrual cycles.

Acupoint location: Acupoint location was established according to the national standard of the People's Republic of China—Name and Location of Acupoints GB/ t12346–2006》.

Needle selection: HUANQIU disposable sterile acupuncture needle, made by Suzhou Acupuncture and Moxibustion Products Co, LTD, has a specification of 0.30×40 mm, which diameter is 0.30 mm and length is 1.5 inches.

Operation method: After the doctor cleans the hands, the patient should be placed in the supine position to expose the skin at the selected acupoint, and local routine disinfection should be conducted after the acupoint selection. The method of lifting, inserting, or twisting is performed after fast insertion, Make patient acupoint feel acid hemp heavy distension, acupuncture doctor hand feels nervous feeling, namely “De Qi.” The abdominal acupoints adopted the method of airflow in and out slowly, and the other acupoints adopted the method of flat supplement and flat purging with the needle kept for 20 minutes.

Operators: At every study hospital, 2 uniformly trained acupuncturists with at least 2 years of clinical experience will participate in the process. The acupuncturist will be provided with treatment protocols, research intent or purpose, treatment records, and training for future monitoring.

### 2.6 The measurement of results

The main outcome indicator was clinical pregnancy rate. Clinical pregnancy rate test method: vaginal b-ultrasonography was performed 5 weeks after transplantation, and clinical pregnancy was diagnosed in patients who saw fetal sac, fetal bud, and fetal heart beat.

Secondary outcome measures were the endometrial 3D volume and blood flow parameters (vascularization index [VI], flow index [FI], and vascularization blood flow index [VFI]), endometrial thickness, endometrial volume, uterine artery PI, RI, and S/D during the “implantation window period” (20–24 days after menstrual cycle of ovary stimulation). The measurement time was before enrollment, after treatment, and 1 day before transplantation. All the patients were scanned by transvaginal 3-dimensional imaging, and the same setting was used. The operation was performed by the same person.

Data were collected from the IVF center database before enrollment, 1 day after treatment and 5 to 40 weeks after transplantation, as well as from patient follow-up. We will collect data on the safety of acupuncture treatment and any adverse events from the treatment of the implementer.

### 2.7 The quality control

To ensure the quality of research, all researchers must attend all training courses and pass the training examinations. They must master all the details of the experiment before they can carry it out. For example, they must master the use of randomization and fill out a Case report form. Acupuncturists should undergo strict and systematic training. The assessor should know how to collect and input data accurately and completely.

To protect confidentiality before, during and after the trial, we will collect, share, and maintain the personal information of potential and registered participants by professionals, and the assessor will pay attention to confidentiality when entering data. During the trial, all details will be recorded, including all adverse events such as needle breakage, bleeding, hematoma, fainting, severe pain, and local infection. Serious adverse events are immediately reported to the principal investigator and immediate rescue and compensation procedures are initiated.

To ensure trial quality, clinical monitors designated by the principal investigator will periodically verify all process details. All operational frequencies and procedures for this study will be reviewed by a professional independent of the investigator and sponsor. If there are significant protocol modifications (e.g., changes to eligibility criteria, outcomes, analyses), the study plan will be communicated to relevant parties (e.g., investigators, REC/IRBs, trial participants, trial registries, journals, regulators).

### 2.8 Statistical analysis

SPSS v. 21.0 Windows was used for statistical analysis. All data are presented as mean ± standard deviation or percentage. Independent samples were compared using the *t* test. Descriptive statistics and bivariate tests (e.g., correlation test, *t* test, and *χ*^2^ test) will be used to summarize the sample demographics and to examine the distribution characteristics and relationships of the study results. Specifically, we will assess the overall difference (group effect) in endometrial three-dimensional volume blood flow parameters (VI, FI, and VFI) between the acupuncture group and the control group, changes during the study period (time effect), and differences in such changes (intergroup interaction) between the groups. Significant group effects or intergroup interactions will indicate significant effects of the intervention.

## 3 Discussion

Endometrial receptivity refers to the state of endometrium in which the embryo attachment, penetration, and implantation are allowed to lead to embryo implantation, and it is an important link to ensure the implantation of pregnant eggs, the development of fetus, and placenta.^[5]^ Studies have shown that the smooth implantation of an embryo is closely related to endometrial receptivity.^[4]^ Chien et al^[12]^ conducted ultrasonic tests on 623 IVF-ET patients and showed that when blood flow was detected in both endometrium and subendometrium, the pregnancy rate and implantation rate increased. Currently, studies have begun to explore whether acupuncture and moxibustion can improve endometrial receptivity. For example, Zheng et al^[13]^ found that percutaneous acupoint electrical stimulation can improve endometrial receptivity, thus increasing the pregnancy rate of IVF-ET patients. Xing et al^[14]^ found that combination of acupuncture and medicine can significantly improve endometrial receptivity of patients. However, there is still insufficient evidence that acupuncture treatment can improve the local microenvironment of endometrium, the endometrial receptivity, and the pregnancy outcome of patients, and a well-designed and standardized multicenter randomized controlled trial is necessary to verify the effectiveness of acupuncture in improving endometrial receptivity and Pregnancy Outcome.

In this study, we choose recurrent implantation failure (RIF), which means that female patients who have had 3 or more failed embryo transplants in the past and who have existing frozen embryos and do not need to have their eggs removed again are the subjects of this study, to rule out the influence of abortion and other factors on endometrial receptivity. The intima threedimensional volumetric blood Flow parameters, including VI (Vascularization index), FI (Flow index) and VFI(vascularized blood Flow index), are taken as the main indexes to reflect the intima tolerance, because we think lining three-dimensional volume under the blood Flow parameters can reflect the endometrium and endometrial regional Vascularization and blood perfusion of the overall situation, can more fully and objectively reflect the endometrium and endometrial blood Flow. At present, there are many factors to evaluate endometrial receptivity, including endometrial thickness, volume, morphology, local endocrine changes, and blood perfusion status. Studies have shown that endometrial volume and endometrial blood flow can more directly reflect the microenvironment of embryo implantation site than uterine artery.^[15]^ Silva and Ginther ^[16]^ found that patients with monitoring of endometrial blood flow had a better implantation rate. Santoshkumar et al's^[17]^ latest study showed that endometrial blood flow could well predict the implantation rate of IVF cycles and was not affected by the morphological manifestations of endometrium. At the same time, we believe that the “sham acupuncture group” is difficult to be truly implemented in clinical practice. Because this study aims to explore the effectiveness of stage-by-stage acupuncture and moxibustion therapy based on the “Chong Channel being sea of blood” theory in improving endometrial receptivity and Pregnancy Outcome after repeated transplantation failure, only the control group and the stage-by-stage acupuncture and moxibustion group based on the “Chong channel being sea of blood” theory were set up.

According to the theory of traditional Chinese medicine, infertility is caused by the mutation of Chong Channel and Ren Channel, dysfunction of Zang-fu organs, and impotence of uterus. Chong Channel as 1 of the 8 abnormal meridians plays an important role in the treatment of infertility. We formed the treatment of female infertility “stage-by-stage acupuncture and moxibustion therapy” based on the 19 years’ clinical practice, on the basis of considering the “Chong Channel being sea of blood” of maintenance of uterus, and the possible correlation between lining and lining under perfusion. In the clinical practice, SP-4(Gongsun) and PC-6(Neiguan) of the 8 pulse intersection points were selected as the main points, and the points were selected according to the menstrual cycle stages and syndrome differentiation of the patients, so as to form the stage-by-stage acupuncture and moxibustion therapy based on the “Chong Channel being sea of blood” theory, and achieved good efficacy in clinical application.

Limitations of this test are all the samples collected are from Asia. Qian et al have shown that the positive effects of acupuncture and moxibustion in Asian regions are stronger than those in non-Asian regions.^[18]^ Therefore, a more comprehensive sample size is needed for comparative analysis.

In conclusion, we conducted a suitable clinical trial design and completed a scientific design combining the characteristics of acupuncture and moxibustion. We will report and analysis clinical trial results ^[19]^ according to the report randomized controlled trials^[20]^ intervention and acupuncture clinical trials reporting standards (acupuncture clinical trials reporting standard intervention) of the international standard. Hope that the results of this study will bring more scientific and rigorous clinical evidence to stage-by-stage acupuncture and moxibustion therapy based on the “Chong channel being sea of blood” theory in IVF - ET failure more than 3 times on the receptivity of endometrium and Pregnancy Outcome, and enrich the treatment methods of IVF-ET.

## 4 Trial status

This trial is currently in the recruitment phase, with recruitment starting on July 20, 2018 and expected to be completed by December 20, 2019. Protocol version number: ChiCTR1800017191. Protocol version date: 2018/07/17.

## Acknowledgments

The authors acknowledged the fund projects that contributed toward the article.

## Author contributions

Zhao Rong, Ma Yanping, Xing Liwei and Xu Jinlong all contributed to developing the study protocol. Xing Liwei and Xu Jinlong drafted the manuscript, and all the authors contributed to writing of the manuscript. All authors read and approved the final version.

**Data curation:** Li Li.

**Investigation:** Haina Zhuang.

**Methodology:** Qiong Zhang, Yunxiu Li, Zhuojun Yuan, Yanping Ma.

**Writing – original draft:** Liwei Xing, Jinlong Xu, Rong Zhao.

**Writing – review & editing:** Liwei Xing, Jinlong Xu.
